# Reprogramming T cell differentiation and exhaustion in CAR-T cell therapy

**DOI:** 10.1186/s13045-023-01504-7

**Published:** 2023-10-25

**Authors:** Yannick Bulliard, Borje S. Andersson, Mehmet A. Baysal, Jason Damiano, Apostolia M. Tsimberidou

**Affiliations:** 1 Appia Bio, 6160 Bristol Pkwy, Culver City, CA 90230 USA; 2 Unit 455, Department of Investigational Cancer Therapeutics, The University of Texas MD Anderson Cancer Center, 1515 Holcombe Blvd., Houston, TX 77030 USA; 3 Department of Stem Cell Transplantation and Cellular Therapy, The University of Texas MD Anderson Cancer Center, 1515 Holcombe Boulevard, Houston, TX 77030 USA

**Keywords:** Differentiation, Exhaustion, Memory, T cell, CAR-T

## Abstract

T cell differentiation is a highly regulated, multi-step process necessary for the progressive establishment of effector functions, immunological memory, and long-term control of pathogens. In response to strong stimulation, as seen in severe or chronic infections or cancer, T cells acquire a state of hypo-responsiveness known as exhaustion, limiting their effector function. Recent advances in autologous chimeric antigen receptor (CAR)-T cell therapies have revolutionized the treatment of hematologic malignancies by taking advantage of the basic principles of T cell biology to engineer products that promote long-lasting T cell response. However, many patients’ malignancies remain unresponsive to treatment or are prone to recur. Discoveries in T cell biology, including the identification of key regulators of differentiation and exhaustion, offer novel opportunities to have a durable impact on the fate of CAR-T cells after infusion. Such next-generation CAR-T cell therapies and their clinical implementation may result in the next leap forward in cancer treatment for selected patients. In this context, this review summarizes the foundational principles of T cell differentiation and exhaustion and describes how they can be utilized and targeted to further improve the design and efficacy of CAR-T cell therapies.

## Introduction

T cells are an essential component of the adaptive immune system, playing a critical role in recognizing and eliminating infected and malignant cells [1]. They differentiate into distinct subsets with specialized functions to enable effective immune surveillance and response. However, prolonged exposure to antigens or chronic infections can lead to T cell exhaustion, which is characterized by diminished functionality and decreased ability to clear target cells [1].

Chimeric antigen receptor (CAR)-T cell therapy is a promising immunotherapeutic approach that involves genetically modifying T cells to express a CAR that recognizes a specific antigen on the surface of cancer cells [2]. CAR-T cell therapy has demonstrated remarkable efficacy in treating hematologic malignancies, with several CAR-T cell therapies currently available for clinical use [3]. In recent years, the US Food and Drug Administration (FDA) has approved six such therapies: Kymriah (tisagenlecleucel), Yescarta (axicabtagene ciloleucel), Tecartus (brexucabtagene autoleucel), Breyanzi (lisocabtagene maraleucel), Carvykti (ciltacabtagene autoleucel), and Abecma (idecabtagene vicleucel). Despite unprecedented benefits to countless patients, autologous CAR-T therapy has not worked equally for all patients with hematologic malignancies. In B cell acute lymphoblastic leukemia (B-ALL) and non-Hodgkin lymphoma (NHL), 50% and 56% of patients, respectively, experienced relapse from CAR-T cell treatment at 12-month follow-up [4, 5], and a cell therapy remains to be approved for chronic lymphocytic leukemia (CLL), where only 26% of patients present with a durable response [6]. CAR-T cell therapy has also not yet shown clinical benefit in a broader range of indications, particularly in solid tumors [7].

The path to broader clinical success of CAR-T cell therapy is likely to involve a series of technological advancements, perhaps even tailored to individual indications, and will certainly incorporate process improvements and engineering techniques meant to control the composition of the product, possibly along the memory-effector and exhaustion axes. Proper understanding of the mechanisms of T cell differentiation and exhaustion and the methods used to manipulate them will be crucial for developing the next generation of CAR-T cell therapies with antitumor efficacy in solid tumors [8]. In this review, we will summarize T cell differentiation and exhaustion processes and the role of T cell biology in CAR-T therapy. We will also provide insights into new therapeutic opportunities.

## Role of T cell differentiation during CAR-T product manufacturing and its impact on clinical outcomes

CAR-T products generated from cancer patients vary widely in terms of differentiation status; such variation is likely a manifestation of differences in the quality of the patients’ starting T cell material [9–14]. In the last couple of years, transcriptomic signatures based on the differential expression of hundreds of memory-, effector-, and dysfunction-associated genes have been used to characterize cell therapies and correlate product characteristics with clinical response. Some of the largest clinical studies published to date have revealed that the clinical efficacy of CAR-T therapy strongly positively correlates with signatures of memory and negatively correlates with signatures of effector function or exhaustion. A study of 41 patients with advanced CLL treated with autologous CAR-T therapy (Kymriah) identified high memory, low effector, and low exhaustion gene scores as determinants of response [12]. Another study of 71 patients with B-ALL and Hodgkin lymphoma (HL) treated with Kymriah identified a network, or regulon, of target genes upregulated by the master regulator of memory function T cell factor-1 (TCF-1) as a strong predictor of response [12, 13]. In a more recent study of 12 acute lymphoblastic leukemia (ALL) patients, the authors used CITE-seq to determine that the frequency of early memory T cells in the CAR-T products of patients treated with Kymriah was predictive of response [15]. In a study of 24 patients with large B cell lymphoma (LBCL) treated with Yescarta, memory CD8 + T cells were significantly more frequent in CAR-T cell products from patients with continued complete response (CR) than those from patients with partial response/progressive disease (PR/PD); the opposite was true for exhausted CD8 + T cells [11]. Intriguingly, an independent study of 32 patients with LBCL found a statistical correlation between a population of memory-like CD8 + T cells and response for patients treated with Kymriah but not for those treated with Yescarta [16]. Finally, in a study of 54 melanoma patients treated with tumor-infiltrating lymphocyte (TIL) therapy, TIL products showing high expression of memory-associated genes and low expression of granzyme A (GZMA) and interferon gamma (IFN-γ) correlated with response [17]. Patients with a complete response received a considerably higher dose of neoantigen-specific TILs with memory characteristics than patients who did not have a response.

While it is now generally well established that CAR-T cell composition has a direct impact on the activity of CAR-T cell therapy in the clinic, a clear definition of the product attributes associated with response remains lacking. While initial attempts to define memory among CAR-T cells relied on historical cell surface markers associated with memory in the peripheral blood, such as L-selectin (CD62L) and chemokine receptor type 7 (CCR7), the direct association between these markers and response has remained elusive and study-dependent. Many studies have not been able to identify a correlation between conventional homing markers and clinical outcome [12, 18–20]. More specifically, CCR7 and CD62L in isolation were not associated with response [12, 20]. When a correlation has been reported, its significance has been relatively weak (*p* = 0.0464 [21], *p* = 0.0317 for central memory T cell (Tcm) [15], and *p* = 0.0327 [22]). At least four clinical trials with a common aim to generate a less-differentiated product from CD62L or CCR7 enrichment in the starting leukapheresis material have yet to report improved persistence and/or response in the clinic (NCT01318317; NCT01815749; NCT02062359). In one study, CAR-T cells generated from CD62L-purified T cells did not show improved efficacy compared to CAR-T cells made from non-enriched T cells (NCT01865617) [23]. Besides enrichment, alterations to the manufacturing process designed to bolster memory in the end product have not yet resulted in clinical activity superior to that of conventional methods (NCT03318900; NCT03274219; NCT01087294).

Shortening the manufacturing process with the intent to improve stemness and potency of the CAR-T product is a tantalizing concept that has seen recent clinical success [24]. A CD19-directed CAR-T therapeutic modality (YTB323), manufactured in less than 2 days, has shown favorable efficacy in 20 patients with B cell lymphoma when treated at a 25-fold lower dose than a more conventional CAR-T therapy [NCT03960840]. Here again, however, correlation between CCR7 expression in the product and response did not appear to be statistically significant. The cause of the discrepancy between the importance of memory in CAR-T product composition and the lack of improved efficacy in products enriched for CD62L or CCR7 may be multifactorial. Of note, the inflammatory cytokine interleukin 12 (IL-12) can induce high levels of CD62L expression despite skewing T cells toward terminal effector differentiation [25]. Furthermore, T cells can be induced in vitro to re-express both CD62L and CCR7 upon T cell receptor (TCR), interleukin-2 (IL-2), or interleukin-21 (IL-21) stimulation [26]. CD27 is a member of the tumor necrosis factor receptor family, which once activated supports memory formation by promoting IL-2-independent survival by maintaining the expression of interleukin-7 receptor-α (IL-7RA) [27, 28]. CD27 was shown in two large studies to correlate with efficacy when co-expressed with CD45RO [12] or CCR7 [11]; thus, CD27 might represent a better predictive tool than the homing markers CD62L and CCR7, when taken in isolation.

## Pharmacokinetic parameters, memory composition, and patient outcomes

Pharmacokinetic parameters such as peak CAR-T cell level in patients, or maximum concentration (Cmax), along with area under the curve (AUC), have been demonstrated to strongly associate with response, irrespective of treatment or indications [4, 12, 22, 29]. However, to date, the connection between product composition and these predictive pharmacokinetic parameters has not been well established. A recent study may shed new light on this fundamentally important question [30]. This study, which used mathematical algorithms trained on existing clinical data to model pharmacokinetic behaviors and predict response based on product characteristics, first found that transcriptomic analysis of the pre-infusion products of two CAR-T products (Yescarta and Kymriah) in three separate indications resulted in more accurate predictions than standard flow-cytometry-based immunophenotyping [30]. Importantly, the authors found that CΑR-T products that were associated with short responses were characterized by deficient proliferative and functional capacity. These qualities are typical of Τ cell exhaustion and terminal differentiation, even within similar memory and effector cell populations. Additionally, CAR-T expansion after product infusion, which drives *C*_max_, is representative of memory T cell proliferative capacity [22].

In other words, CAR-T product effectiveness is likely dictated not only by population frequencies but also by the cells’ proliferative capacity, a feature intrinsically associated with the early differentiation of T cells. Given the importance of the composition of CAR-T products for clinical efficacy, it becomes essential to understand T cell differentiation in a broader biological context.

## T cell differentiation during acute response to antigens

**Fig. 1:**
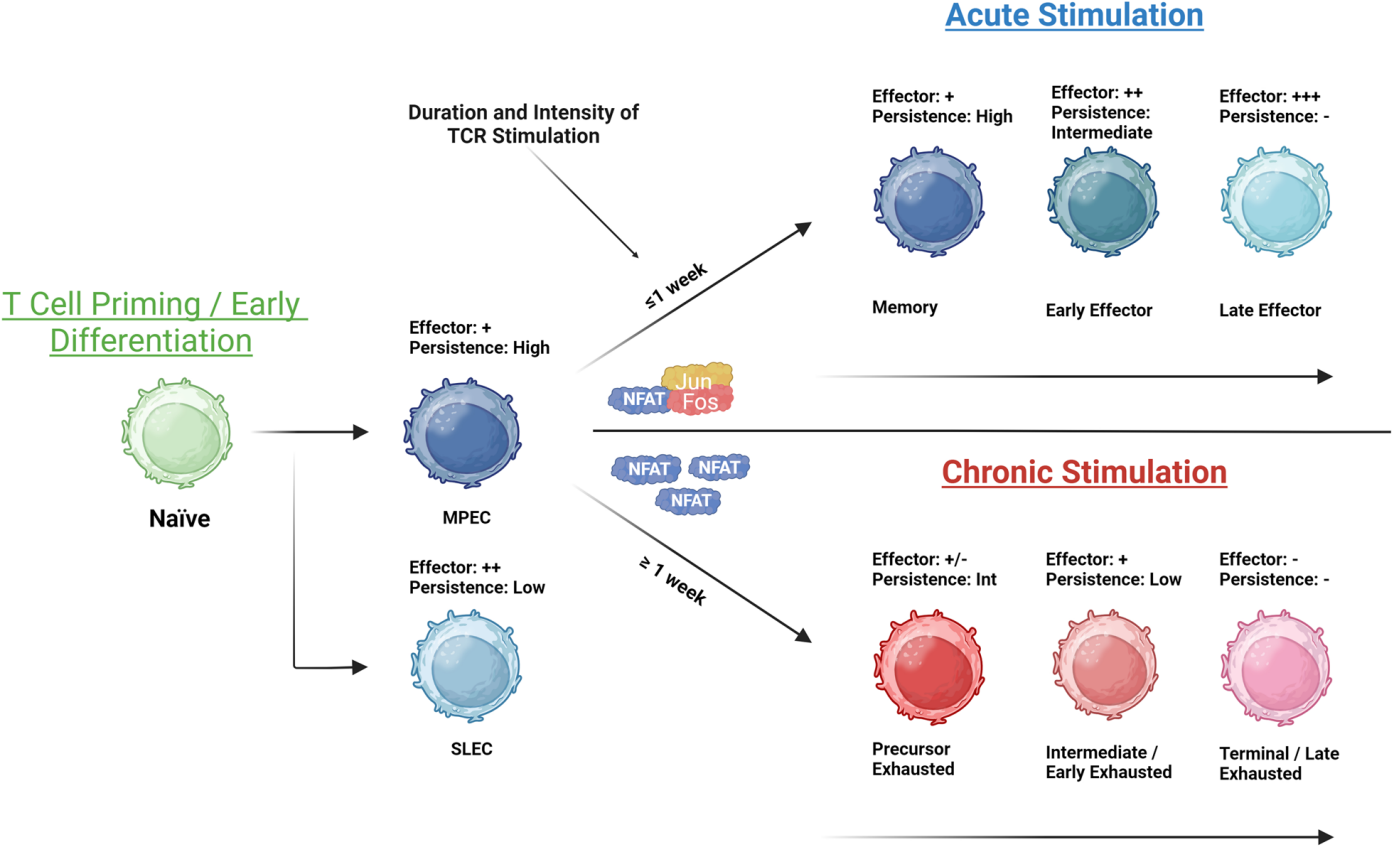
CD8 + T cell differentiation model in acute and chronic antigenic stimulation settings. Peerless NFAT appears to be one of the key upstream determinants of the differentiation path taken by T cells. “Created with BioRender.com”. SLEC: short-lived effector cells

T cell differentiation is a highly regulated process initiated the moment a T cell encounters its cognate antigen. During the ensuing expansion phase, the immune response is dominated by effector T cells (Teff) that mediate antigen clearance, followed by a contraction phase where Teff give way to long-lived memory T cells (Tmem) [31]. Tmem cells uniformly retain high developmental and proliferative potential compared to Teff cells, which is facilitated by their ability to metabolically recruit additional capacities (e.g., high spare respiratory capacity) [31]. Memory T cells that home to secondary lymphoid organs or circulate in the periphery can be identified by enhanced expression of IL-7RA, which allows them to undergo homeostatic proliferation in the absence of TCR stimulation. Depending on their localization, memory T cells also express high levels of homing molecules, such as CD62L, CCR7 in lymphoid organs, CX3CR1 in the periphery, and CD103 and Hobit in tissues [32]. As T cells undergo the process of differentiation, they progressively acquire hallmarks of effector function, such as increased cytotoxic activity and cytokine production, while progressively losing their ability to proliferate (Fig. 1).

## T cell differentiation and exhaustion during chronic antigen response

The above-described T cell differentiation, including the formation of memory T cell subsets, is driven by the eradication of the antigen and thus the resolution of the infection. When a disease and its associated antigens persist at high levels, as seen in chronic infections or cancer, T cells undergo an alternative T cell differentiation path commonly known as T cell exhaustion. Exhausted T cells (Tex) are defined by sustained expression of inhibitory receptors, also known as checkpoint inhibitors, such as programmed cell death protein 1 (PD-1), lymphocyte activation gene 3 (LAG-3), 2B4, and cytotoxic T-lymphocyte-associated antigen 4 (CTLA-4); a diminished ability to produce effector cytokines such as IFN-γ and tumor necrosis factor (TNF); and a reduced ability to proliferate [33]. They are also characterized by high activity of TCR-responsive transcription factors, including thymocyte selection-associated high-mobility group box (TOX), B cell-activating transcription factor (BATF), interferon regulatory factor 4 (IRF4), and nuclear factor of activated T cell (NFAT) [34]. This phenotypic and transcriptional profile has recently been extended to demonstrate that Tex cells fundamentally differ from regular T cells based on a distinct epigenetic landscape [35–37]. T cells in chronic infection and in tumors acquire exhaustion-specific patterns of chromatin accessibility [38–41]. Tmem cells have an open chromatin structure specifically enriched for binding sites to basic leucine zipper, interferon regulatory factors, and T-box transcription factors (TFs). In contrast, Tex cells (like Teff cells) harbor an open chromatin structure around effector genes; however, Tex cells are uniquely enriched for binding sites to NFAT and nuclear receptor subfamily 4 group A (NR4A) [42], and effector genes like IFN-γ show high levels of negative DNA methylation in line with their hypofunctional state [42, 43]. Importantly, this pattern is stably imprinted and cannot be reversed by the blockade of immune checkpoint inhibitors such as with the anti-PD-1 antibodies used in the clinic [43, 44].

Similar to effector T cell differentiation during acute stimulation, under chronic stimulation T cells undergo sequential differentiation toward terminal exhaustion (Fig. 1). An early form of dysfunctional T cell, herein referred to as precursor exhausted T cells (Tpex), was first identified in a model of chronic viral infection [45]. In mice, Tpex cells are detected within the first week after the initial challenge, indicating that commitment to a hypo-responsive state takes up to 7 days of continuous TCR stimulation [46]. Similar to peripheral Tmem cells, Tpex cells express TCF-1, retain a self-renewing capacity, and can differentiate into effector-like Tex cells. However, Tpex cells also express TFs associated with differentiation, such as TOX, nuclear factor of activated T cells 2 (NFATC2), and BATF [47], and several checkpoint inhibitors, such as PD-1 and T cell immunoreceptor with Ig and ITIM domain (TIGIT) [48]. Tpex are also limited in their proliferation and cytokine production capacity [48]. In line with other types of Tex cells, Tpex cells are epigenetically locked in an exhausted state, without the ability to return to a more physiological state [44]. In human cancer, higher levels of TCF-1 + Tpex cells in tumors have been linked to longer patient survival and improved therapeutic outcomes and are essential for the long-term maintenance of T cell response [49–53]. Similarly, Tpex cells have been shown to play an important role in the therapeutic activity of antibodies blocking checkpoint inhibitors [49–51, 53, 54], highlighting the importance of this T cell subset for immunity in both humans and mice.

The continuous presence of antigens leads to the emergence of terminally differentiated and exhausted T cells (Tex^term^). Trajectory analyses support a model whereby Tex^term^ cells are derived from Tpex cells [55]. Tex^term^ cells are identified by the high expression of eomesodermin (EOMES), TOX, CD69, and checkpoint inhibitors such as PD-1, LAG-3, or T cell immunoglobulin domain and mucin domain 3 (TIM-3) and low levels of T-BET [56]. During progression toward late dysfunction, traditional effector properties such as the capacity to produce interleukin-2 (IL-2), TNF, and IFN-γ fade away and eventually disappear entirely [57]. Tex^term^ cells correlate with more severe disease in HIV and are enriched in lung tumor tissue compared with blood [58]. Interestingly, in an autochthonous model of lung cancer, most intratumoral T cells eventually differentiate into Tex^term^ cells, and this shift coincides with progression of the tumor from a “hot,” or T cell inflamed, microenvironment to a “cold,” non-T cell inflamed, state [59], suggesting that, given enough time, T cells continually exposed to TCR stimulation terminally differentiate and become inoperant.

## Regulation of T cell differentiation during acute and chronic conditions

T cell differentiation during acute and chronic stimulation alike is a gradual process that is tightly regulated by epigenetic and transcriptional modulators, whose abundance and timing of expression is key to proper fate commitment. Interestingly, although chronic and acute stimulation results in vastly different outcomes, the types of modulators involved in each process overlap considerably.

### Transcriptional regulation of differentiation in the context of acute antigenic challenge

**Fig. 2:**
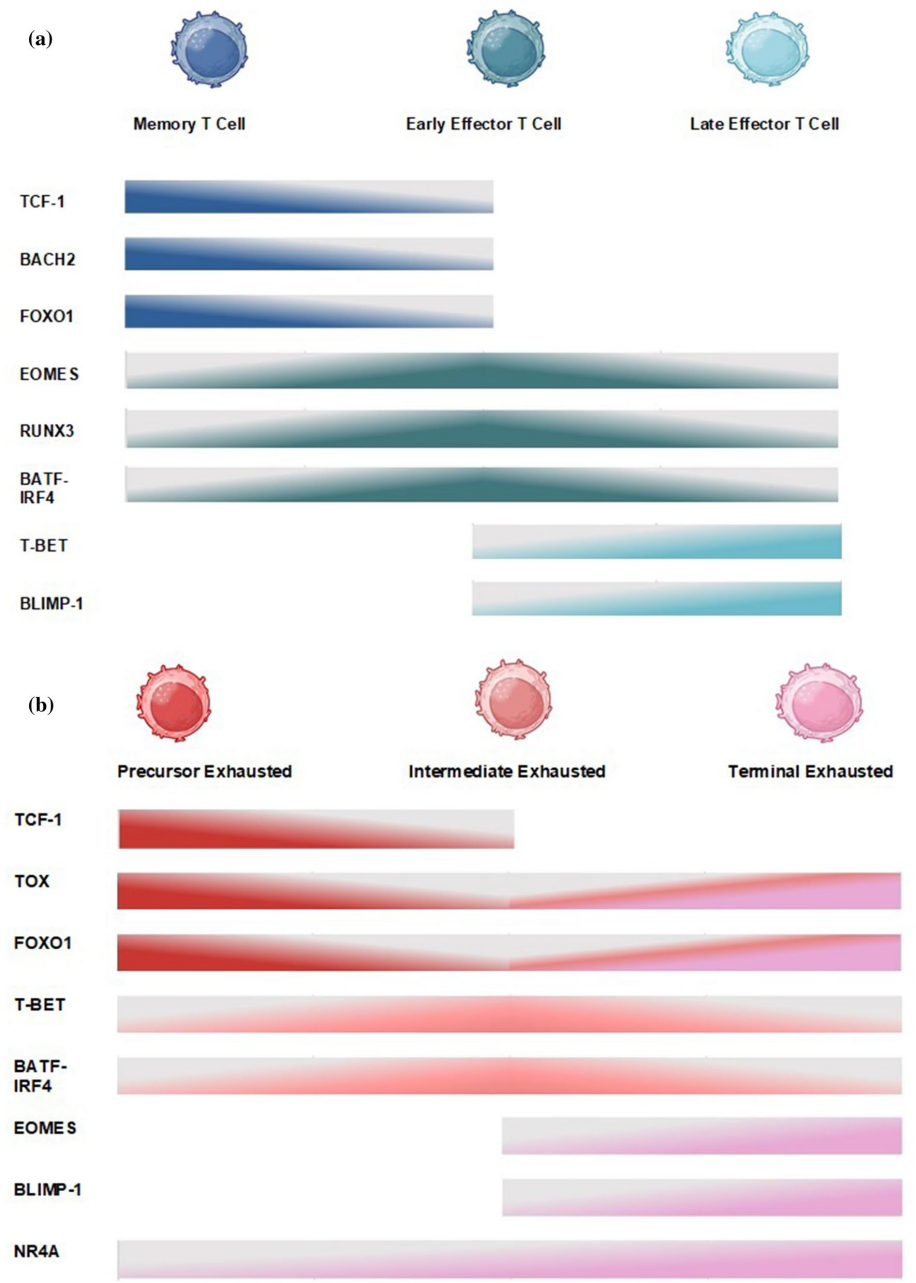
Graded expression of key regulators of T cell differentiation and exhaustion in acute and chronic conditions. **A**: During acute TCR stimulation, the following factors are highest among Tmem cells and gradually decrease in expression during the process of effector differentiation: TCF-1 [80], BACH2 [77], and FOXO1 [75]. Expression of EOMES [81], RUNX3 [82], and BATF-IRF4 [83, 84] is low among Tmem cells, peaks among early Teff cells and recedes in late Teff cells. Finally, the following factors gradually increase in expression and contribute to effector T cell differentiation: T-BET [85] and BLIMP-1 [86]. **B**: During chronic TCR stimulation, the following factors are highest among Tpex cells and gradually decrease in expression along the course of exhaustion: TCF-1 [35, 87], TOX [87, 88], and FOXO1 [35, 73, 76]. While TCF-1 remains low, expression of TOX and FOXO1 gradually increases as T cells become terminally exhausted. On the other hand, expression of T-BET [85, 87, 89], BATF-IRF4 [35, 90], and NR4A [91] is at its lowest in Tpex cells and is induced during chronic TCR engagement. While T-BET and BATF-IRF4 expression peaks among intermediate exhausted T cells and subsequently recedes, NR4A expression remains high and peaks at the Tex^term^ cell stage. Expression of EOMES [87, 89, 92] and BLIMP-1 [93] also peaks at the terminal exhausted stage, but their gradual increase in expression starts later, at the intermediate exhausted stage

In the early stages of an infection, most T cells differentiate toward an effector function (short-lived effector cells, or SLECs), whereas a subset of activated T cells (memory precursor effector cells, or MPECs) are poised to become long-term, self-renewing memory T cells, protecting the host from recurring infections. At single-cell resolution, the transcriptional profile of CD8 + T cells at the onset of TCR engagement shows striking divergence and reveals two distinct subpopulations along a memory-effector axis after the first division [60–62]. The canonical Wnt-signaling pathway is important for the maturation and homeostasis of peripheral memory T cells [63], and the Wnt-dependent factor TCF-1 and its functional homolog lymphoid enhancer-binding factor-1 (LEF-1) are key regulators in the formation of memory and the inhibition of effector differentiation [64, 65] (Fig. 2A). TCF-1 utilizes both transcriptional regulation and histone deacetylation via its intrinsic HDAC domain to direct T cell fate [66, 67]. EOMES acts directly downstream of TCF-1 [65] and is critical to maintain memory, in part because it promotes sustained expression of interleukin-2 receptor subunit beta (IL-2RB), thus supporting interleukin-15 (IL-15) and IL-2 signaling and continued proliferation [68, 69]. Forkhead O transcription factor 1 (FOXO1) enforces stem cell-like properties and represses T-BET, IFN-γ, and granzyme B (GZMB) effector functions [70–73], and its continuous activity is necessary for the maintenance of memory in both acute and chronic conditions [74–76]. FOXO1 acts upstream of TCF-1, as it directly binds and upregulates expression of the TF [70, 75]. TCF-1 and FOXO1 act in synergy by promoting expression of the pro-memory and pro-survival genes EOMES, IL-7RA, CD62L, CCR7, and B cell leukemia 2 (BCL-2) [71, 73, 75]. Inversely, FOXO1 is inhibited by mammalian target of rapamycin complex 1 (mTORC1), which is in line with the opposing effects of FOXO1 and mammalian target of rapamycin (mTOR) signaling on CD8 + T cell differentiation [71]. BACH2, another downstream target of FOXO1, establishes a stem-like transcriptional program at the single-cell level [77] and contributes to memory formation by restricting the access of JUN family TFs to the regulatory elements of TCR-induced genes [78]. Interestingly, BACH2 also enacts an epigenetic program of memory, with reduced chromatin accessibility at regions open in terminally exhausted CD8 + T cells and genomic regions controlled by TFs such as runt-domain transcription factors (RUNX) and BATF. BACH2 promotes inhibitor of DNA-binding 3 (ID3) and B cell lymphoma 6 (BCL-6) expression but suppresses the expression of killer cell lectin-like receptor subfamily G member 1 (KLRG1) and B-lymphocyte-induced maturation protein 1 (BLIMP-1) [79].

Likewise, downstream differentiation is tightly regulated by its own set of transcriptional and epigenetic modulators. In CD8 + T cells, the interleukin-21 (IL-21)- and interleukin-12 (IL-12)-induced TF BATF regulates chromatin accessibility and promotes the upregulation of key transcriptional regulators of effector differentiation such as runt-domain transcription factor 3 (RUNX3), T-BET, and BLIMP-1 and, together with IRF4 and Jun, upregulates the IL-12 and IL-2 signaling pathways [83, 94, 95]. On the other hand, BATF represses expression of the effector molecules IFN-γ and GZMB. Hence, BATF augments the propagation of inflammatory signals while restraining expression of downstream effector molecules, thus acting as a gateway in the differentiation process. RUNX3, another BATF-induced TF [83], is essential for long-term immunity [96]. RUNX3 governs chromatin accessibility to a broad number of cis-regulatory regions and upregulates IRF4 and BLIMP-1, while it mediates downregulation of the memory TFs BACH2 and TCF-1. Similar to BATF, RUNX3 curtails terminal differentiation by limiting T-BET expression, thus ensuring timely expression of regulators of differentiation. The inflammatory cytokine IL-12 directs further differentiation by inducing expression of the effector TFs T-BET and BLIMP-1 and downregulating factors essential for memory-like TCF-1 and IL-7RA [80, 97]. T-BET favors the induction of a terminally differentiated state [97] and, together with BLIMP-1, promotes the expression of effector molecules such as granzymes while repressing memory-associated factors such as TCF-1, IL-7RA, and CD62L [98]. BLIMP-1 has a negative effect on proliferation, and its expression correlates with a greater level of apoptosis after restimulation [99, 100]. Type I interferons induce expression of the NOTCH family of receptors and, together with interleukin-2 receptor (IL-2R), mTOR, and T-BET, form a positive feedback module of differentiation that integrates signals from various sources [101]. Altogether, it is the graded expression of competing sets of transcription factors that controls CD8 + T cell differentiation and fate commitment toward memory versus effector T cell function.

### Transcriptional regulation of differentiation in the context of chronic antigen stimulation

During chronic antigen stimulation, the induction of exhaustion is independent of inflammatory signals and other environmental factors; instead, the degree of exhaustion is directly linked to the quantity of antigen present as well as the duration/frequency of TCR stimulation [40, 102]. It should be noted that, contrary to exhaustion, other forms of T cell dysfunction can be induced by the tumor microenvironment, and factors such as tumor-associated hypoxia, acidity, and altered lipid metabolism can impair CAR-T cell functionality, while the presence of immunosuppressive cell populations can curtail their activity. This important topic has been extensively discussed elsewhere [103–106]. The balance between NFAT cells and activator protein 1 (AP-1) dictates the outcome of the branching point between physiological versus hypo-responsive differentiation, and chronic TCR stimulation drives this imbalance, resulting in “partnerless” NFAT [107], which directs a transcriptional and epigenetic program of exhaustion [42, 108] (Fig. 1). Partnerless NFAT induces a transcriptional program characterized by continuous expression of the repressive transcription factors early growth response 2 (EGR2), zinc finger protein 2 (IKZF2), IRF4, TOX, and NR4A and other exhaustion-associated factors [108–112]. Downstream of these initiating events, many of the same regulatory factors found during acute stimulation play a similar role in the chronic setting (Fig. 2B). For instance, TCF-1 and FOXO1 both play a central role in the generation and maintenance of Tpex cells [64, 65, 73, 74, 113, 114]. FOXO1 directly promotes expression of PD-1, which, in a positive feedback loop, indirectly promotes expression of FOXO1 via inhibition of mTOR signaling [73]. Likewise, BACH2 is required for long-term immunity in chronic viral infection [115], while BLIMP-1 antagonizes memory formation in both chronic (Tpex) and acute (Tcm) conditions [93, 99]. BLIMP-1 expression is highest in the highly differentiated Tex^term^ [93]. BATF together with IRF4 cooperates to establish exhaustion [116]. The NFAT-induced regulator TOX is highly expressed among Tex cells in both humans and mice, particularly in Tpex and Tex^term^ cells [87, 88, 111, 117]. TOX directly contributes to the open chromatin structure and continuously high levels of expression of multiple checkpoint inhibitors, including PD-1 [20, 87, 88, 111]. TOX binds to chromatin remodeling factors and thus contributes to the exhaustion-linked epigenetic landscape of Tex cells [88, 111]. The role of TOX in T cell dysfunction may be time dependent, since short-term expression of TOX is not sufficient to induce terminal exhaustion [88, 111]. In sequence with TOX, the NR4A family of TFs contributes to T cell exhaustion by orchestrating a genome-wide, exhaustion-linked alteration of the epigenetic and transcriptomic landscape in CD8 + TILs and CAR-T cells [112, 118]. Like TOX, NR4A TFs directly bind to and upregulate PD-1 gene expression [112]. NR4A1 represses effector gene expression by inhibiting AP-1 function, and its ablation enhances antitumor immunity [91]. Interestingly, NR4A1 plays a central role in general tolerance since its ablation exacerbates autoimmunity in a model of induced colitis [91].

## Controlling CAR-T differentiation via cell engineering

The discrepancy between the clinical benefit of high memory composition in CAR-T cells and the absence of demonstrated improvement of CAR-T cells generated under memory-skewing conditions is puzzling. One confounding factor is that markers typically used as surrogates for memory, such as CD62L or CCR7, might not be appropriate markers for in vitro expanded CAR-T cells. However, it is also quite plausible that to increase efficacy over current CAR-T therapies and to demonstrate durable clinical benefit a regulatory network of memory needs to be stably engineered to retain memory characteristics post-infusion. Accordingly, we will focus this section on engineering approaches meant to stably reprogram CAR-T cells’ differentiation and exhaustion states. Note that other recent reviews have addressed the question of next-generation CAR-T enhancements more broadly, beyond manipulation of differentiation and exhaustion [119, 120].

Factors involved in T cell memory or dysfunction that have been evaluated in the context of adoptive cell therapy are summarized in Table 1. One precursor study revealed that constitutive expression of BCL-6, a master regulator of T follicular helper CD4 + T cell differentiation and repressor of BLIMP-1 [121], can increase homeostatic proliferation and the maintenance of memory T cells, which increases their frequency over time [122]. The potential to directly modulate transcription factors in CAR-T cells specifically was more recently demonstrated via ectopic expression of the AP-1 transcription factor cJUN [123]. Using three different CAR constructs against as many targets, Lynn and colleagues demonstrated that ectopically expressed cJUN improves the antitumor activity as well as persistence of CAR-T cells in vivo. cJUN may possibly compete for chromatin binding with NR4A3, which is known to repress the development of memory T cells [124]. In contrast, a subsequent mouse study failed to show any benefit of ectopic expression of cJUN; instead, co-expression of cJUN with BATF reduced the advantage conferred by ectopic expression of BATF alone [84]. However, in a subsequent study using exhaustion-inducing conditions (i.e., low effector-to-target ratios) BATF deletion, not overexpression, improved the antitumor immunity conferred by CAR-T cells, both in vitro and in vivo [125]. Alternatively, the improved antitumor activity associated with cJUN overexpression might be due to an improved ability of T cells to recognize low levels of antigens [126]. The master transcriptional regulator of central memory, TCF-1, is another interesting choice for CAR-T cell therapies. Murine T cells expressing TCF-1 ectopically show greater control of chronic viral infection and vastly out-compete wild-type T cells 30–40 days post-infection [67]. In a B16 melanoma model, adoptive transfer of TCF-1-positive T cells mediated greater tumor control, lower degrees of differentiation and exhaustion, and consequently greater cytokine production than their wild-type counterparts [65]. Natural killer T (NKT) cells are a type of αβ T cells endowed with innate immune properties. LEF-1, a functional homolog to TCF-1, promotes expansion when ectopically expressed in NKT cells transduced with an anti-GD2 CAR construct and improves antitumor efficacy in vivo [127]. In a chronic model of viral infection, deletion of FOXO1 leads to a decrease in memory T cells and loss of viral control over time [73], making FOXO1 a prime candidate to improve the stemness of a CAR-T product. Inducible expression of a constitutively active form of FOXO1, as well as TCF-1, after adoptive transfer of CAR-T cells leads to increased persistence compared to control CAR-Ts [128]. BACH2, another master regulator of stemness, has not been investigated in the context of CAR-T cells; however, in a model of chronic viral infection, enforced expression of BACH2 in adoptively transferred CD8 + T cells promotes the establishment of a memory transcriptional program and induced prolonged persistence of T cells in the absence of differentiation or exhaustion [79]. The same study revealed that ectopic expression of another TF implicated in the regulation of survival and stemness, SRY-Box transcription factor 4 (SOX4), increases memory recall response [79]; however, its potential role as an oncogene severely limits its use in cell therapy [129]. In an adoptive transfer model, enforced expression of RUNX3 improves the overall survival of B16-bearing mice and increases the accumulation of T cells over time within the tumor environment, possibly by limiting the degree of terminal differentiation/exhaustion [130]. Interestingly, RUNX3 also enforces a tissue-resident memory phenotype and function and promotes localization to the small intestine endothelium in mice, a distinctive feature that could be exploited for the treatment of colorectal cancer, for instance [130].

**Table 1:** List of factors involved in T cell memory or dysfunction evaluated in the context of adoptive cell transfer

Factor	Type	Adoptive transfer setting	Functional improvement in vivo (overexpression/downregulation)	References
*Positive regulators of stemness for overexpression strategy in CAR-T therapy*				
BCL-6	Zinc finger C2H2-type BTB domain-containing TF	OVA-specific murine CD8 + T cell	Improved memory response over 4 weeks (OVA-pulsed DC) or 10 weeks (VV-OVA)	[122]
cJUN	Basic leucine zipper TF	CD19, CD22, HER2, and GD2-specific human CAR-T cells	Improved intratumoral T cell function and efficacy, particularly in low antigen density setting	[123]
		Murine CD19 CAR-T cells	No improvement compared to unmodified CAR-T cells	[84]
TCF-1	TCF/LEF TF	GP33-specific murine TCR-T cells	Greater levels of cytokine production, improved antitumor response and recall response	[131]
		HER2-specific CAR-T cells	Improved expansion upon rechallenge	[128]
FOXO1	Forkhead boxes TF	HER2-specific CAR-T cells	Improved expansion upon rechallenge	[128]
BATF	Basic leucine zipper TF	Murine CD19 CAR-T cells	Improved intratumoral CAR cytokine production, decreased expression of checkpoint inhibitors and TOX, increased memory response	[84]
LEF-1	TCF/LEF TF	GD2-specific CAR-T cells	Improved antitumor response and survival	[127]
BACH2	BTB domain-containing, basic leucine zipper TF	SIY peptide-specific murine TCR-T cells	Improved antigen recall response	[79]
SOX4	SRY-box TF	SIY peptide-specific murine TCR-T cells	Improved antigen recall response	[79]
RUNX3	Runt-related TF	GP33-specific murine TCR-T cells	Improved antitumor response and survival	[130]
*Positive regulators of dysfunction for downregulation strategy in CAR-T therapy*				
BATF	Basic leucine zipper TF	Mesothelin-specific CAR-T cells	Improved antitumor response and survival. Increased tumor infiltration, IFN-γ production and proliferation	[125]
DNMT3A	DNA/RNA methyltransferase	EphA2, HER2 and IL-13Rα2-specific CAR-T	Improved antitumor response and survival, longer persistence and greater multipotency index	[132]
SUV39H1	Lysine methyltransferase	OVA-specific murine CD8 + T cells	Improved recall response to secondary bacterial infection	[133]
NR4A1	Nuclear receptor subfamily 4 group A	OVA-specific murine CD8 + T cells	Improved antitumor response, increased IFN-γ production, and decreased expression of TIM-3 and PD-1	[91]
NR4As	Nuclear receptor subfamily 4 group A	CD19-specific murine CAR-T cells	Improved antitumor response, increased IFN-γ production, and decreased expression of TIM-3 and PD-1	[118]
TOX + TOX2	Thymocyte selection-associated high-mobility group box TFs	CD19-specific murine CAR-T cells	Improved antitumor response and survival, increased IFN-γ and TNF production, and decreased expression of TIM-3, PD-1, and LAG-3	[112]
TOX	Thymocyte selection-associated high-mobility group box TFs	GP33-specific murine TCR-T cells	No pharmacokinetic difference during acute infection. Rapid decline of blood T cell during chronic infection, despite lower level of checkpoint inhibitor expression. Lower levels of TCF-1 Monoallelic TOX deletion improves antitumor activity	[111]
		TAG-epitope-specific murine TCR-T cells	No functional or phenotypic difference after immunization Decreased infiltration of liver tumor and increased levels of apoptosis, despite lower level of checkpoint inhibitor expression	[88]
		GP33-specific murine TCR-T cells	Increased response to acute infection (transient). Survival deficit in the blood during chronic infection. Lower frequency of TCF-1 + T cells	[134]
BLIMP-1 + NR4A3	BLIMP-1: PR/SET domain family, zing fingers C2H2-type NR4A3: Nuclear receptor subfamily 4 group A	PSMA-specific CAR-T cells	Improved antitumor response and survival, increased IFN-γ, TNF, and IL-2 production, increased TCF-1 and decreased TIM-3 and PD-1 expression	[93]
ARID1A	BAF complex, AT-rich interaction domain containing	OVA- and B7-H3-specific murine CAR-T cells *ARID1A was blocked with a small molecule inhibitor	Improved antitumor response and survival and increased persistence of CD62L + T cells at day 30	[135]
		OVA-specific murine CD8 + T cells	Improved antitumor response and survival	[136]
HPK1	Mitogen-activated protein 4 kinase	CD19-, HER2-, and BCMA-specific CAR-T cells	Improved antitumor response and survival, greater tumor infiltration, increased expression of CD107a, TNF and granzyme B, and decreased expression of PD-1, TIM-3, and LAG-3	[137]
BTG1	BTG/Tob family	GD2-specific CAR-NKT cells	Improved antitumor response and survival	[138]
PD-1	V-set domain-containing immunoglobulin	CD19-, HER2-, and BCMA-specific CAR-T cells	Improved antitumor response and survival, greater tumor infiltration, increased expression of CD107a, TNF, and granzyme B, and decreased expression of PD-1, TIM-3, and LAG-3	[137]
		GP33-specific murine T cells	Decreased IFN-γ and TNF expression after stimulation with LCMV clone 13 (chronic model of viral infection) and diminished long-term proliferation	[139]

These modulators represent engineering modifications of potential interest for CAR-T cell therapeutics, with the common aim to stably imprint memory function or limit dysfunction or exhaustion

The suppression of factors driving the exhaustion of T cells is another approach that has shown promising results in preclinical models. Recently, deletion of DNA methyltransferase 3A (DNMT3A) was shown to preserve the antitumor activity of CAR-T cells during prolonged tumor exposure [132]. This was accompanied by a lower methylation profile; increased expression of TCF-1, LEF-1, and CCR7; sustained cytokine secretion; and cytolytic activity upon repeat antigenic challenge. CAR-T cells depleted of DNMT3A were able to control a tumor rechallenge 148 days after the initial tumor challenge, which is indicative of long-term memory. Interestingly, a retrospective analysis of CAR-T cells in CLL revealed a significantly lower expression score for genes targeted by DNMT3A in the products from non-responders, compared to all other groups, in support of the negative role of this factor in CAR-T antitumor potency [12, 132]. Another epigenetic regulator of differentiation, histone lysine methyltransferase (SUV39H1), is involved in the silencing of stem/memory genes, and its depletion increased survival and long-term memory in a model of bacterial infection [133].

Two downstream targets of NFAT have also received recent attention: the NR4A family of orphan nuclear receptors and the TOX family of (high-mobility group box) DNA-binding proteins. Deletion of NR4A1 [91] and all three NR4A family members [118] confers robust antitumor responses to CD8 + tumor-infiltrating T lymphocytes by restoring AP-1 function. Likewise, ablation of TOX and TOX2 in adoptively transferred T cells confers almost complete immunity to an aggressive model of melanoma [112]. An important caveat here is that, under chronic conditions, TOX is required for the long-term maintenance of immunity [88, 111, 134], which makes the applicability of TOX deletion for CAR-T cell therapy questionable, especially in the context of chronic antigenic exposure, which may occur within the microenvironment of epithelial tumors. Likewise, while heterozygous ablation of BLIMP-1 increases expression of CD62L and TCF-1 on T cells [93, 140], complete abrogation of BLIMP-1 results in reduced cytotoxic activity and loss of immunity in a chronic model of viral infection [93]. This issue can be circumvented by co-down-regulation of NR4A3, a compensatory mechanism upregulated upon BLIMP-1 deletion [141], or by targeting a positive regulator of BLIMP-1 expression, hematopoietic progenitor kinase-1 (HPK1) [137]. Two groups found that a member of the chromatin remodeling cBAF complex, AT-rich interactive domain-containing protein 1A (ARID1A), was a negative determinant of Tmem cell fate and promoted the acquisition of exhaustion-associated features, respectively [135, 136]. Pharmacological or genetic ablation of ARID1A resulted, in both cases, in improved in vivo antitumor function. BTG anti-proliferation factor 1 (BTG1), a cell stress regulator, has been found to be essential for establishing exhaustion in a murine model of chronic viral infection [142]. Interestingly, it is also associated with exhaustion among CAR-NKT cells in the clinic, and BTG1 downregulation in these cells improves expansion and in vivo antitumor functionality [138].

Functional ablation of PD-1 in human CAR-T cells confers improved antitumor activity in various solid tumor xenograft models [143–145]. During chronic viral infection, complete PD-1 removal initially increases the proliferation of antiviral T cells during the expansion phase [139]; however, during the ensuing contraction phase, PD-1 ablation precipitates the natural decline of antiviral T cells, ultimately resulting in fewer memory cells [139, 146]. These data bring to light the duality of T cell dysfunction in cell therapy, increasing the risk for tumor immune escape due to T cell hypo-functionality but also maintaining long-term immunity in conditions of chronic stimulation. The right balance may depend on the relative tumor burden [22], with a high tumor burden leading to more prolonged antigen exposure and a more profound exhaustion state of the cell therapy product [30].

Collectively, the above data suggest that in patients with solid tumors or hematologic malignancies alike, CAR-T cells would be more successful if used earlier in the course of the disease. Instead of being used as primary salvage therapy in high-risk patients, they should be developed as maintenance therapy for patients whose disease has responded to initial therapy and who have a decreased tumor volume.

## Conclusions

Despite profound clinical successes in defined and relatively small patient populations, the full potential of engineered T cell therapies across the larger oncology landscape has yet to be realized. Numerous innovative approaches are being leveraged to deliver a new generation of medicines with the potential to overcome many of the hurdles that are believed to be responsible for the limited efficacy of CAR-T cell therapy related to premature T cell exhaustion, particularly in solid tumors. The modulation of gene transcription programs responsible for differentiation and exhaustion in T cells, via ectopic expression or ablation of epigenetic regulators or transcription factors, is a promising approach to provide more durable antitumor efficacy than has been possible with unmodified CAR-T cells. Through these genetic manipulations, a slew of next-generation CAR-T cells have been designed to engage tumor targets for a longer duration while counteracting the negative effect of terminal differentiation and/or exhaustion, showing improved efficacy in preclinical models. Because of the stable nature of transcriptional modifications, these functional memory “armored” T cell therapies have the potential to provide a more durable clinical benefit than previous approaches, and perhaps even overcome the limitations of functional cell persistence that may be responsible for failures in treating solid tumors. At the forefront of this new generation of therapeutics, a first-of-its-kind clinical study is investigating the antitumor potential of LYL797, a ROR1-targeted CAR-T cell therapy armored with cJun (NCT05274451), while a second study is using mesothelin-targeted CAR-T cells armored with a dominant-negative form of the checkpoint receptor PD-1 (NCT04577326).

## Future perspectives

Besides using blood-derived T cells as starting material in the manufacture of CAR-T cells, other cell subsets may offer distinct, unique advantages. The use of CD4 + T cells, innate-like T lymphocytes, as well as cord blood-derived hematopoietic stem cells to generate next-generation CAR-based therapeutic modalities is particularly tantalizing.

### *CD4* + *T cells*

Despite extensive research into the functionality of CD8 + T cells, mounting evidence suggests that other T cell subsets, in particular CD4 + T cells, may also contribute to the efficacy of CAR-T or TCR-T therapies. When combined with CD8 + T cells, CD4 + T cells with an under-differentiated phenotype dramatically improved the therapeutic activity of adoptively transferred T cells in preclinical tumor models [147, 148]. In addition to providing helper functions, CD4 + CAR-T cells can elicit potent cytotoxic activity, are less susceptible to activation-induced cell death, and express lower levels of inhibitory immune checkpoint receptors compared to CD8 + CAR-T cells [148, 149]. Interestingly, native cytolytic CD4 + T cells may be important for antitumor control in human bladder cancer [150, 151]. Altering T cell polarization toward helper T cells is another tantalizing approach to enhance the activity of CAR-T therapies. GATA3, a master regulator of Th2 T cells, is positively correlated with long-term persistence of CAR-T cells in humans [13]. A composite score of activity along the IL-6/STAT3 pathway, a key pathway of Th2 differentiation among CAR-T cells, has been correlated with positive clinical responses [12]. Finally, CAR-T cells generated under Th9-polarizing conditions have also been explored as a means of improving therapeutic activity. Th9 T cells express high levels of IL9, TNFA, and IL-2 and lower levels of IFNG; they also exhibit a less-differentiated phenotype compared to conventional CAR-T cells after prolonged culture in vitro [152]. In a humanized mouse model, these Th9-polarized CAR-T cells have demonstrated a tangible improvement in efficacy compared to conventional CAR-T cells [152].

### Innate-like T lymphocytes

As an alternative to conventional CD4 + or CD8 + T cells, a new class of engineered cell therapies exploiting the favorable biological features of innate-like T lymphocytes is in development. *γδ* T cells and NKT cells express an invariant TCR and may be engineered to express CARs to target specific tumor-associated antigens [153–156]. Unlike CAR-T cells, NKT and *γδ* T cells express highly functional activating NK receptors that endow them with the ability to kill tumor cells in the absence of expression of CAR- or TCR-targeting antigens [157]. This feature enhances the potential of engineered invariant T cells to effectively eradicate heterogeneous tumors expressing variable levels of CAR-targeting antigens and to mitigate antigen escape as a resistance mechanism. Other investigators have found that CAR- or TCR-engineered NKT cells may modulate the immunosuppressive microenvironment in syngeneic mouse tumor models by minimizing the presence of myeloid-derived suppressor cells and enhancing the presence of pro-inflammatory macrophages [158]. Through the cross-priming of dendritic cells, adoptively transferred engineered NKT cells have also been demonstrated to enhance the antitumor efficacy of host CD8 + cytotoxic T cells, inducing durable protection against tumor rechallenge [159]. Thus, NKT cells have the potential to overcome many of the persistent inhibitory signals in the tumor microenvironment that promote exhaustion and limit the functional activity of CAR-T cells in cancer types that have proven to be unsusceptible to treatment. To further enhance the ability of NKT and T cells to avoid exhaustion in the face of chronic antigen stimulation or inhibitory signals from the tumor microenvironment, IL-15 armoring has been demonstrated to promote the self-renewal of progenitor exhausted cells [160, 161].

### Cord blood-derived hematopoietic stem cells

A fundamental challenge of autologous CAR-T cell therapy, only partially addressed by the next-generation advancements reviewed here, is driven by the vast patient-to-patient differences in the quality of blood-derived T cells [12]. Similarly, non-genetically modified tumor-infiltrating lymphocytes, expanded in vitro for therapeutic application, have been found to be typically hypofunctional and differentiated [162]. Healthy donor-derived, allogeneic, “off-the-shelf” cell therapies are now being developed to alleviate this specific issue [163]. An important challenge of off-the-shelf CAR-T therapies manufactured from blood-derived mature T cells is the need to generate large batches of cells via extensive ex vivo expansion, which may limit their proliferative capacity and functionality in patients. New manufacturing techniques have been deployed to address this potential issue and allow CAR-T or CAR-NKT cells to maximize their capacity for expansion and persistent tumor cell killing in vivo [155, 164]. To this end, the use of umbilical cord blood-derived CD34 + hematopoietic stem cells transduced with a non-alloreactive iNKT TCR as starting material presents several unique advantages [155]. It alleviates the need to suppress expression of the endogenous TCR in an allogeneic context; furthermore, it allows for the large expansion of cells during manufacture, without leading to terminal differentiation of the end product, since a majority of the expansion phase occurs prior to the emergence of mature, functional T cells.

Although the technological advances cited here have the potential to rectify premature exhaustion and inadequate antitumor efficacy of cell therapies, definitive validation data from clinical studies are likely years away. Other strategies to curb the negative influence of the tumor microenvironment, tumor-associated hypoxia, acidity, altered lipid metabolism, and metabolomics are fields of ongoing research to advance CAR-T cell functionality. Thus, the study of additional T cell-enhancing approaches in the clinic is needed to ensure that the next generation of therapies becomes highly effective and potentially curative drugs for a wider population of patients than is currently being served by the approved autologous T cell products in hematological malignancies and emerging data from solid tumors.

## Data Availability

Not applicable.

## Abbreviations

ALL: Acute lymphoblastic leukemia
AP-1: Activator protein 1
ARID1A: AT-rich interactive domain-containing protein 1A
AUC: Area under the curve
B-ALL: B cell acute lymphoblastic leukemia
BATF: B cell-activating transcription factor
BCL-2: B cell leukemia 2
BCL-6: B cell lymphoma 6
BLIMP-1: B-lymphocyte-induced maturation protein 1
BTG1: BTG anti-proliferation factor-1
CAR-T: Chimeric antigen receptor T cell
CCR7: Chemokine receptor type-7
CD103: Cluster of differentiation-103
CD62L: L-selectin
CLL: Chronic lymphocytic leukemia
Cmax: Maximum concentration
CR: Complete response
CTLA-4: Cytotoxic T-lymphocyte-associated antigen-4
CX3CR1: Motif chemokine receptor-1
DNMT3A: DNA methyltransferase 3A
EGR2: Early growth response 2
EOMES: Eomesodermin
FDA: Food and Drug Administration
FOXO1: Forkhead O transcription factors-1
GATA3: GATA-binding protein-3
GvHD: Graft-versus-host disease
GZMA: Granzyme A
GZMB: Granzyme B
HPK1: Hematopoietic progenitor kinase-1
ID3: Inhibitor of DNA-binding-3
IFN-γ: Interferon gamma
IKZF2: Zinc finger protein 2
IL-12: Interleukin-12
IL-15: Interleukin-15
IL-2: Interleukin-2
IL-21: Interleukin-21
IL-2R: Interleukin-2 receptor
IL-2RB: Interleukin-2 receptor subunit beta
IL-6: Interleukin-6
IL-7RA: Interleukin-7 receptor-Α
IL9: Interleukin-9
IRF4: Interferon regulatory factor 4
IRFs: Interferon regulatory factors
KLRG1: Killer cell lectin-like receptor subfamily G member 1
LAG-3: Lymphocyte activation gene 3
LBCL: Large B cell lymphoma
LEF-1: Lymphoid enhancer-binding factor-1
MPEC: Memory precursor effector cell
mTOR: Mammalian target of rapamycin
mTORC1: Mammalian target of rapamycin complex 1
NFAT: Nuclear factor of activated T cell
NFATC2: Nuclear factor of activated T cells 2
NHL: Non-Hodgkin lymphoma
NKT: Natural killer T cells
NR4A: Nuclear receptor-4A
NR4A1: Nuclear receptor-4A1
NR4A3: Nuclear receptor-4A3
NR4As: Nuclear receptor subfamily 4 group A
PD: Progressive disease
PD-1: Programmed cell death protein 1
PR: Partial response
RUNX: Runt-domain transcription factors
RUNX3: Runt-domain transcription factors-3
SOX4: SRY-box transcription factor 4
SLEC: Short-lived effector cell
STAT3: Signal transducer and activator of transcription-3
SUV39H1: Histone lysine methyltransferase
TCF-1: T cell factor-1
Tcm: Central memory T cell
TCR: T cell receptor
Teff: Effector T cells
Teff: Effector T cells
Tex: Exhausted T cells
Texterm: Terminally differentiated and exhausted T cells
TF: Transcription factor
TIGIT: T cell immunoreceptor with Ig and ITIM domain
TIL: Tumor-infiltrating lymphocytes
TIM-3: T cell immunoglobulin domain and mucin domain 3
Tmem: Memory T cells
TNF: Tumor necrosis factor
TNFA: Tumor necrosis factor alpha
TOX: Thymocyte selection-associated high-mobility group box
Tpex: Precursor exhausted T cells
